# The validity of self-reported number of teeth and edentulousness among Norwegian older adults, the HUNT Study

**DOI:** 10.1186/s12903-022-02116-2

**Published:** 2022-03-21

**Authors:** Hedda Høvik, Marit Kolberg, Linda Gjøra, Line Cathrine Nymoen, Rasa Skudutyte-Rysstad, Lene Hystad Hove, Yi-Qian Sun, Tone Natland Fagerhaug

**Affiliations:** 1 Center for Oral Health Services and Research, Mid-Norway (TkMidt), Trondheim, Norway; 2 The Norwegian National Centre for Ageing and Health, Vestfold Hospital Trust, Tønsberg, Norway; 3 Department of Psychiatry, Levanger Hospital, Nord-Trøndelag Hospital Trust, Levanger, Norway; 4 Oral Health Centre of Expertise in Eastern Norway, Oslo, Norway; 5 Department of Cariology and Gerodontology, Faculty of Dentistry, University of Oslo, Oslo, Norway; 6 Department of Clinical and Molecular Medicine, Faculty of Medicine and Health Sciences, Norwegian University of Science and Technology (NTNU), Trondheim, Norway; 7 Department of Public Health and Nursing, Faculty of Medicine and Health Sciences, Norwegian University of Science and Technology (NTNU), Trondheim, Norway

**Keywords:** Epidemiology, Self-report, Number of teeth, Tooth count, Edentulous, Oral health, Validity, Cognitive function, Older adults

## Abstract

**Background:**

Number of teeth is an established indicator of oral health and is commonly self-reported in epidemiological studies due to the costly and labor-intensive nature of clinical examinations. Although previous studies have found self-reported number of teeth to be a reasonably accurate measure, its accuracy among older adults ≥ 70 years is less explored. The aim of this study was to assess the validity of self-reported number of teeth and edentulousness in older adults and to investigate factors that may affect the accuracy of self-reports.

**Methods:**

This study included two different samples of older adults ≥ 70 years drawn from the fourth wave of the Trøndelag Health Study (the HUNT Study), Norway. Sample 1 (n = 586) was used to evaluate the validity of self-reported number of teeth and sample 2 (n = 518) was used to evaluate self-reported edentulousness. Information on number of teeth and background variables (education, smoking, cognitive function, and self-perceived general and oral health) were self-reported in questionnaires, while clinical oral health examinations assessed number of teeth, number of teeth restored or replaced by fixed prosthodontics and edentulousness. Spearman and Pearson correlation coefficients, Bland–Altman plot, chi-square test and kappa statistics were used to assess the agreement between self-reported and clinically recorded number of teeth.

**Results:**

The mean difference between self-reported and clinically recorded number of teeth was low (− 0.22 teeth), and more than 70% of the participants reported their number of teeth within an error of two teeth. Correlations between self-reports and clinical examinations were high for the total sample (0.86 (Spearman) and 0.91 (Pearson)). However, a lower correlation was found among participants with dementia (0.74 (Spearman) and 0.85 (Pearson)), participants having ≥ 20 teeth (0.76 (Spearman) and 0.67 (Pearson)), and participants with ≥ 5 teeth restored or replaced by fixed prosthodontics (0.75 (Spearman) and 0.77 (Pearson)). Self-reports of having teeth or being edentulous were correct in 96.3% of the cases (kappa value 0.93, *p* value < 0.001).

**Conclusions:**

Among older Norwegian adults, self-reported number of teeth agreed closely with clinical tooth counts and nearly all the edentulous participants correctly reported having no teeth.

## Background

Number of remaining natural teeth is a widely used indicator for oral health as the predominant cause of tooth loss is oral diseases, i.e., caries and periodontitis [1, 2]. This indicator is used in epidemiological studies both to investigate oral health [3] and to explore possible associations between oral conditions and other diseases [4–6]. Tooth loss is related to higher risks of systemic disease and mortality [7, 8], as well as reduced quality of life [9].

Using clinical examinations to record number of teeth and other dental parameters in population-based studies is both time-consuming and labor-intensive, and often expensive. In addition, a clinical oral examination may be burdensome for persons with impaired health and persons of older age. An easily available alternative is to use self-reported number of teeth, applied in questionnaires or interviews, and hence more suitable in larger epidemiological studies. Several studies have used self-reported measures to explore oral health status at group- or population level [10–12]. Knowing the validity and reliability of self-reported values is therefore crucial. Recent studies exploring the validity of self-reported number of teeth have found a high correlation between self-reported and clinical tooth counts at group level [13–16].

The population is rapidly aging worldwide [17]. Although increased life expectancy implies benefits both on the individual and societal level, aging also involves gradual physical and cognitive decline and many older people live with disabilities in the last years of their life [18]. Regarding oral health, aging predicts fewer remaining teeth [19]. Among older adults, tooth loss is a robust measure for oral health status and is strongly associated with general health and mortality [7, 8, 20]. Moreover, aging naturally affects the dental condition through wear-and-tear and oral diseases, resulting in a lower number of sound teeth and more prosthodontic restorations compared to the younger population. Increased complexity of the dental status may increase the risk of miscounting the number of natural teeth in self-reports [21–24]. Furthermore, dementia is known to affect several cognitive domains, like calculation, executive functions, attention, and memory [25], which may challenge the accuracy of self-reported number of teeth. In addition, self-reported measures can be influenced by age, socio-economic, cultural, environmental, and other individual factors. Consequently, the use of self-reported data requires an updated evaluation of their validity in different populations. However, few studies have investigated the accuracy of self-reported number of teeth in older populations [23, 24].

The aim of this study was to assess the validity of self-reported number of present natural teeth and self-reported edentulousness in Norwegian older adults. We also wanted to investigate possible differences between the subjective and objective measures in relation to demographic, socio-economic, lifestyle, cognitive function, and general and oral health factors.

## Methods

In this study, we used data from two different samples drawn from the fourth wave of the Trøndelag Health Study (HUNT). HUNT invited all adult residents in the northern part of Trøndelag County, Norway, to participate in four comprehensive surveys, the HUNT1 Survey (1984–1986), the HUNT2 Survey (1995–1997), the HUNT3 Survey (2006–2008) and the HUNT4 Survey (2017–2019). The HUNT Study comprises questionnaires, clinical measurements, and collections of biological samples [26, 27]. In HUNT4, the entire adult population from 23 municipalities in the northern part of Trøndelag county was invited to participate.

**Fig. 1:**
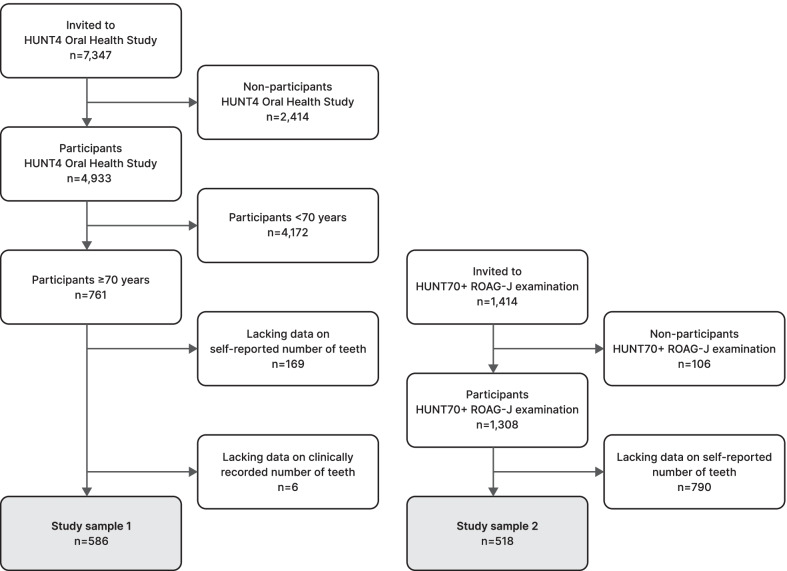
Inclusion and exclusion criteria of study samples 1 and 2

Study sample 1 consisted of older adults examined at HUNT4 field stations. A random sample (n = 7,347) from six municipalities were invited to an oral health examination at field stations (HUNT4 Oral Health Study). A total of 4,933 participants were examined, of whom 761 were 70 years and older. Among these, there was no information on self-reported (n = 169) or clinical (n = 6) number of teeth in 175 participants. Our final study sample 1 consisted of 586 older adults (Fig. 1). This sample was used to evaluate the validity of self-reported number of teeth.

Study sample 2 consisted of older adults examined in their homes or at nursing homes. This sample was based on the HUNT70 + study part of HUNT4. A total of 1414 adults 70 years and older were invited to an oral health examination in the participant’s home, or at the nursing home, of whom 1308 persons accepted to participate. Among these, there was no information on self-reported number of teeth in 790 participants. Our final study sample 2 consisted of 518 older adults (Fig. 1). This sample was used to evaluate the validity of self-reported edentulousness.

### Ethical considerations

The HUNT4 Survey including the HUNT4 Oral Health Study and HUNT70 + was approved by the Norwegian Data Protection Authority. Informed consent was obtained from all participants and/or their legal guardians. The current study was performed in accordance with relevant guidelines and regulations and was approved by the Norwegian Regional Committees for Medical and Health Research Ethics (81406/REK midt) and the Norwegian Centre for Research Data (NSD 155523). The study was performed in accordance with the Declaration of Helsinki.

### Self-reported number of teeth

Information on self-reported number of teeth was obtained through a questionnaire asking participants to respond to the following question: “How many of your own teeth do you still have?”. No guidance or further instructions on how to count their teeth were given. In study sample 2, all respondents reporting zero number of teeth were defined as being edentulous.

### Clinically recorded number of teeth and edentulousness

For study sample 1, clinical and radiographic examinations were performed by trained and calibrated dentists and dental hygienists at HUNT4 field stations following a standard protocol (HUNT4 Oral Health Study). The radiographic examinations included orthopantomogram and bitewing radiographs. The variables used from the clinical examination included number of present teeth (hereafter termed clinical number of teeth) and number of teeth restored or replaced by fixed prosthodontics (i.e., crowns, implants, bridge abutments and pontics). In total, 32 participants had root remnants (27 participants had one, three had two, and two had three root remnants). Root remnants were not included in the variable clinical number of teeth used in the analyses. The variable clinical number of teeth included third molars and primary teeth.

For study sample 2, the Revised Oral Assessment Guide-Jönköping (ROAG-J) was part of the examinations conducted by nursing staff at nursing homes and home visits (HUNT70+). The nursing staff received training in ROAG-J from dental personnel prior to the examinations. ROAG-J is a standardized instrument to be used by non-dental professionals to assess and document oral health status and problems in older persons [28]. The instrument includes the recording of the presence of own teeth (yes/no). This variable was used in study sample 2 for comparison of self-reported and clinically recorded edentulousness. However, counting the number of present natural teeth was not part of the examination.

### Covariates

Data on education, smoking status, and self-perceived general and oral health were self-reported in the questionnaires. The participants’ level of education was dichotomized into primary/secondary school (≤ 12 years) and university/college (> 12 years). Smoking status was reported as never / former daily / former occasionally / current daily / current occasionally and was dichotomized into never smokers or ever smokers.

The question for self-perceived general health was: “How is your health at the moment?” with responses: very good / good / not so good / poor*.* We combined very good / good into good and not so good / poor into poor*.* The question for self-perceived oral health was: “How would you say your dental health is?” with responses: very good / good / poor / very poor. The information on self-perceived oral health was dichotomized into good or poor.

Cognitive function was evaluated by a battery of assessment tools and questionnaires, as described by Gjøra et al. [29]. Based on the available data, medical physicians with clinical and scientific expertise diagnosed the participants with no cognitive impairment, amnestic mild cognitive impairment, non-amnestic mild cognitive impairment, or dementia [29]. In the current study the information on cognitive function was grouped into no cognitive impairment/mild cognitive impairment/dementia, with amnestic and non-amnestic mild cognitive impairment combined in the category mild cognitive impairment.

### Statistical analysis

The validity of self-reported number of teeth was evaluated by comparing self-reported to clinical number of teeth in study sample 1. The Bland–Altman plot was used for investigating the overall agreement between self-reported and clinical number of teeth by plotting the mean difference of the two measures. The 90% and 95% limits of agreement were calculated as ± 1.65 and ± 1.96 standard deviations (SD). In addition, the paired t-test was used to test the difference between self-reported and clinical number of teeth. The correlation between the two tooth count measures was analyzed by applying Spearman and Pearson correlation coefficients, both for the whole study sample and among different subgroups: sex, age, education, smoking status, cognitive function, general and oral health, number of teeth, and number of teeth restored or replaced by fixed prosthodontics. In addition, we performed a chi-square test for trend and linear regression adjusted for age and sex to investigate the relationship between number of teeth restored or replaced by fixed prosthodontics and self-reported error. The validity of self-reported edentulousness was evaluated by kappa statistics comparing self-reported to clinical number of teeth in study sample 2. Statistical analyses were performed using IBM® SPSS Statistics 27.0.1.

## Results

In study sample 1, the mean age of the total population was 75.6 (standard deviation (SD) 4.6) years (Table 1). Close to one-fifth of the study population was 80 years or older and there was an equal distribution of females and males. About two-thirds of the study population were current or former smokers. No cognitive impairment was reported for 57.8% of the participants, 26.3% were diagnosed with mild cognitive impairment and 5.5% were diagnosed with dementia, while information on cognitive function was unknown for 10.4% of the participants. Two-thirds of the population reported their general health to be good and about 80% reported their oral health to be good. Clinical examinations showed that the mean number of present teeth was 21.0 (SD 7.2), less than 4% were edentulous and the mean number of teeth restored or replaced by fixed prosthodontics was 5.4 (SD 5.1). About half of the population had five or more teeth restored or replaced by fixed prosthodontics.

**Table 1:** Characteristics of participants in study samples 1 and 2

	Study sample 1 (n = 586)	Study sample 2 (n = 518)
Sex, n (%)		
Female	294 (50.2)	357 (68.9)
Male	292 (49.8)	161 (31.1)
Age, mean (SD)	75.6 (4.6)	86.4 (6.9)
Age, n (%)		
70–74 years	320 (54.6)	34 (6.6)
75–79 years	157 (26.8)	69 (13.3)
80 + years	109 (18.6)	415 (80.1)
Education, n (%)		
Primary/secondary school (≤ 12 years)	378 (64.5)	335 (64.7)
College/university (> 12 years)	205 (35.0)	34 (6.6)
Unknown^a^	3 (0.5)	149 (28.8)
Smoking status, n (%)		
Never smokers	210 (35.8)	170 (32.8)
Ever smokers	370 (63.1)	217 (41.9)
Unknown^a^	6 (1.0)	131 (25.3)
Cognitive function, n (%)		
No cognitive impairment	339 (57.8)	67 (12.9)
Mild cognitive impairment	154 (26.3)	141 (27.2)
Dementia	32 (5.5)	288 (55.6)
Unknown^a^	61 (10.4)	22 (4.2)
Self-perceived general health, n (%)		
Good	396 (67.6)	96 (18.5)
Poor	184 (31.4)	285 (55.0)
Unknown^a^	6 (1.0)	137 (26.4)
Self-perceived oral health, n (%)		
Good	473 (80.7)	311 (60.0)
Poor	84 (14.3)	138 (26.6)
Unknown^a^	29 (4.9)	69 (13.3)
Clinical number of teeth, mean (SD)	21.0 (7.2)	–
Clinical number of teeth, n (%)		
≤ 19 teeth	162 (27.6)	–
≥ 20 teeth	424 (72.4)	–
Self-reported number of teeth, mean (SD)	20.8 (7.9)	8.8 (9.8)
Edentulousness, n (%)		
Clinically recorded edentulous	22 (3.8)	217 (41.9)
Self-reported edentulous	24 (4.1)	220 (42.5)
Fixed prosthodontics^b^, mean (SD)	5.4 (5.1)	
Fixed prosthodontics^b^, n (%)		
None	113 (19.3)	–
1–2 teeth	98 (16.7)	–
3–4 teeth	93 (15.9)	–
5 or more teeth	282 (48.1)	–

*SD* standard deviation

^a^Missing information is coded as unknown

^b^Teeth restored or replaced by fixed prosthodontics (i.e., crowns, implants, bridge abutments and pontics)

The population in study sample 2 (Table 1) was on average ten years older than the population in study sample 1. The mean age was 86.4 (SD 6.9) years and 80.1% of the population was 80 years or older. There were more than twice as many females than males. About one-fourth of the population had mild cognitive impairment (27.2%) and more than half had dementia (55.6%). More than half of the participants (55.0%) reported poor general health and one-fourth (26.6%) reported poor oral health. At the clinical examination, 42.5% of the participants were identified as edentulous.

**Fig. 2:**
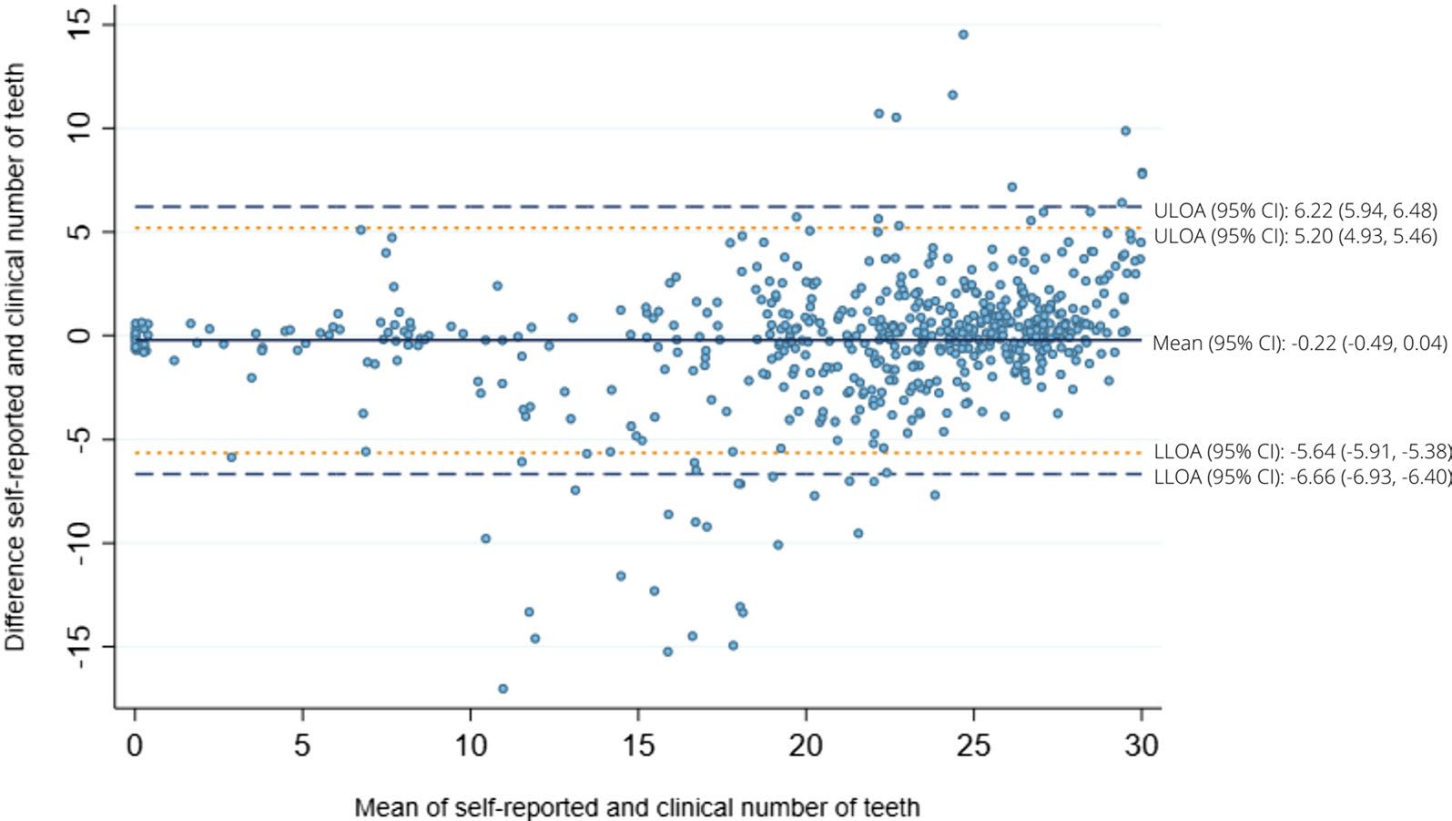
Bland–Altman plot of the agreement between self-reported and clinical number of teeth in study sample 1 (n = 586). Solid dark blue line: mean difference. Dashed blue line: mean difference ± 1.96 standard deviations. Dashed orange line: mean difference ± 1.65 standard deviations. *ULOA* upper limit of agreement, *LLOA* lower limit of agreement, *CI* confidence interval

The Bland–Altman plot (Fig. 2) displays the difference between self-reported and clinical number of teeth (y-axis) to the mean of the two tooth count measures (x-axis). Participants reported on average 0.22 teeth less than clinical number of teeth indicating a slight self-report underestimation. The paired t-test did not show a difference (95% CI: − 0.49 to 0.04, *p* value 0.10). The limits of agreement were relatively wide, with an upper and lower limit of agreement from 6.22 to − 6.66 with ± 1.96 SD and from 5.20 to − 5.64 with ± 1.65 SD, representing 95% and 90% of the expected differences, respectively. The plot also illustrated that the over- and underestimation were extreme in some of the cases.

When root remnants were included in the variable clinical number of teeth, the mean difference between self-reported and clinical number of teeth was increased from − 0.22 teeth (95% CI: − 0.49 to 0.04) to − 0.29 teeth (95% CI: − 0.56 to − 0.02). In supplementary analyses only including the 32 participants with root remnants, the mean difference increased from − 0.44 teeth (95% CI: − 1.53 to 0.65) to − 1.66 teeth (95% CI: − 2.75 to − 0.56) when root remnants were included, confirming more self-report underestimation among those with root remnants.

In all, 35% of the participants had complete agreement between the self-reported and the clinical number of teeth (Table 2). More than 70% of the participants were within a self-report error of two teeth and close to 90% within a self-report error of four teeth. Slightly more participants overestimated (34.5%) than underestimated (30.6%) the number of own teeth.

**Table 2:** Proportion of participants and levels of self-report error by tooth count difference in study sample 1 (n = 586)

Tooth count difference	Overestimation, n (%)	Underestimation, n (%)	Total self-report error, n (%)
0	–	–	205 (35.0)
1	83 (14.2)	55 (9.4)	138 (23.5)
2	43 (7.3)	36 (6.1)	79 (13.5)
3	33 (5.6)	23 (3.9)	56 (9.6)
4	21 (3.6)	23 (3.9)	44 (7.5)
5	8 (1.4)	9 (1.5)	17 (2.9)
≥ 6	14 (2.4)	33 (5.6)	47 (8.0)
Total	202 (34.5)	179 (30.6)	586

Table 3 shows the correlation between clinical and self-reported number of teeth for the whole sample and for subgroups in study sample 1. For the total sample, Spearman and Pearson correlation coefficients were 0.86 and 0.91, respectively, indicating a high correlation between the two measures. Consistently with this, there was also a high correlation between the two measures for both females and males and according to age group, educational level, smoking status, and self-perceived general and oral health. Participants with dementia displayed slightly lower correlation coefficients, 0.74 (Spearman) and 0.85 (Pearson), than participants with no cognitive impairment, 0.87 (Spearman) and 0.91 (Pearson). Participants with ≥ 20 teeth had lower correlation coefficients, 0.76 (Spearman) and 0.67 (Pearson), than those with ≤ 19 teeth, 0.90 (Spearman) and 0.90 (Pearson). The highest correlation coefficient was found among those with no teeth restored or replaced by fixed prosthodontics, 0.98 (Spearman) and 0.99 (Pearson). The correlation coefficients decreased considerably with increasing number of teeth restored or replaced by fixed prosthodontics, with the lowest correlation observed for five or more teeth by fixed prosthodontics, 0.75 (Spearman) and 0.77 (Pearson).

**Table 3:** Correlation of clinical and self-reported number of teeth in study sample 1 (n = 586)

	Clinical number of teeth		Self-reported number of teeth		Spearman^a^	95% CI	Pearson^a^	95% CI
	Median (IQR)	Mean (SD)	Median (IQR)	Mean (SD)				
Total	23 (19–26)	21.0 (7.2)	23 (18–26)	20.8 (7.9)	0.86	0.84–0.88	0.91	0.90–0.92
Sex								
Female	23 (19–26)	21.1 (7.2)	23 (18–26)	20.7 (7.8)	0.88	0.85–0.91	0.93	0.91–0.94
Male	23 (18.5–26)	20.9 (7.2)	23 (18–26)	20.8 (8.0)	0.84	0.81–0.88	0.90	0.87–0.92
Age								
70–74 years	25 (21–27)	22.7 (6.0)	24 (20–27)	22.6 (6.7)	0.83	0.80–0.87	0.90	0.87–0.92
75 + years	21 (16–25)	18.9 (8.0)	21 (12–25)	18.5 (8.7)	0.87	0.84–0.90	0.91	0.89–0.93
Education^b^								
Primary/secondary school	22 (17–25)	19.8 (7.7)	22 (16–26)	19.6 (8.2)	0.88	0.85–0.90	0.93	0.91–0.94
College/university	25 (21–27)	23.3 (5.5)	25 (20–27)	22.9 (6.8)	0.81	0.75–0.85	0.85	0.81–0.84
Smoking status^b^								
Never smokers	24 (21–26)	22.6 (5.6)	24 (20–26)	22.6 (6.3)	0.81	0.76–0.85	0.87	0.84–0.90
Ever smokers	23 (17–26)	20.2 (7.7)	22 (15–26)	19.9 (8.5)	0.88	0.85–0.90	0.92	0.90–0.93
Cognitive function^b^								
No cognitive impairment	24 (20–26)	21.7 (6.8)	24 (19–27)	21.2 (7.5)	0.87	0.85–0.90	0.91	0.89–0.93
Mild cognitive impairment	23 (19–26)	20.7 (7.2)	23 (18–26)	20.8 (8.2)	0.84	0.78–0.88	0.90	0.86–0.93
Dementia	21 (13.5–23)	17.6 (8.0)	20 (10.5–23.5)	17.3 (8.6)	0.74	0.52–0.87	0.85	0.71–0.92
Self-perceived general health^b^								
Good	24 (20–26)	21.7 (6.7)	24 (19–27)	21.5 (7.5)	0.86	0.83–0.88	0.91	0.89–0.92
Poor	23 (17–25)	19.5 (8.1)	21 (14–26)	19.1 (8.6)	0.87	0.83–0.90	0.92	0.89–0.94
Self-perceived oral health^b^								
Good	24 (20–26)	22.3 (6.2)	24 (20–27)	22.2 (7.0)	0.83	0.80–0.86	0.88	0.86–0.90
Poor	18 (8–22.5)	15.5 (7.9)	15 (8–21)	14.4 (8.0)	0.91	0.86–0.94	0.92	0.88–0.95
Clinical number of teeth								
≤ 19 teeth	14 (6–17)	11.4 (6.7)	11 (5–18)	11.5 (7.7)	0.90	0.86–0.92	0.90	0.86–0.92
≥ 20 teeth	25 (23–27)	24.6 (2.5)	25 (22–27)	24.3 (4.3)	0.76	0.72–0.80	0.67	0.61–0.72
Fixed prosthodontics^c^								
None	21 (4–26)	15.8 (10.9)	20 (4–26)	16.1 (11.2)	0.98	0.97–0.99	0.99	0.99–1.00
1–2 teeth	25 (21–27)	23.1 (6.1)	26 (21–27)	23.2 (6.4)	0.91	0.87–0.94	0.95	0.93–0.97
3–4 teeth	24 (21–26)	22.9 (4.8)	24 (20–27)	23.2 (5.3)	0.83	0.75–0.88	0.87	0.80–0.91
5 or more teeth	23 (19–25)	21.7 (5.1)	22 (18–26)	21.0 (6.6)	0.75	0.69–0.80	0.77	0.72–0.82

*CI* confidence interval, *IQR*: interquartile range, *SD* standard deviation

^a^All *p* values < 0.001

^b^Missing information for education (n = 3), smoking status (n = 6), cognitive function (n = 61), self-perceived oral health (n = 29), self-perceived general health (n = 6)

^c^Teeth restored or replaced by fixed prosthodontics (i.e., crowns, implants, bridge abutments and pontics)

We observed a trend of an increasing number of restored or replaced teeth with more self-report error (*p* value < 0.001 for chi-square test for trend), as shown in Table 4. The proportion of participants with self-report error increased with increasing number of teeth restored or replaced by fixed prosthodontics. Among those who misreported their number of teeth, three out of four had three or more teeth restored or replaced by fixed prosthodontics. In addition, linear regression analysis adjusted for age and sex showed that one unit increase in number of teeth restored or replaced by fixed prosthodontics was associated with a self-reported error of 0.22 (absolute value, 95% CI: 0.18 to 0.26, *p* value < 0.001).

**Table 4:** Comparison of self-report error and number of teeth restored or replaced by fixed prosthodontics in study sample 1 (n = 586)

Number of teeth restored or replaced by fixed prosthodontics^a^, n (%)	Self-report error, n (%)		
	None	± 1 or more teeth	Total
None	74 (36.1)	39 (10.2)	113 (19.3)
1–2 teeth	44 (21.5)	54 (14.2)	98 (16.7)
3–4 teeth	22 (10.7)	71 (18.6)	93 (15.9)
≥ 5 teeth	65 (31.7)	217 (57.0)	282 (48.1)
Total	205	381	586

Chi-square test for trend, *p* value < 0.001

^a^Crowns, implants, bridge abutments and pontics

Table 5 compares self-report and clinically recorded edentulousness in study sample 2. Most participants (96.3%) correctly reported having teeth or being edentulous (Table 5). The agreement was almost perfect with kappa value 0.93 (*p* value < 0.001) [30].

**Table 5:** Comparison of self-reported and clinical edentulousness in study sample 2 (n = 518)

Self-reported, n (%)	Clinical examination, n (%)			
	Dentulous	Edentulous		Total
Dentulous	290 (96.3)	8 (3.7)		298 (57.5)
Edentulous	11 (3.7)	209 (96.3)		220 (42.5)
Total	301	217		518

Kappa 0.93, *p* value < 0.001

## Discussion

This is the first study evaluating the validity of self-reported number of teeth among a larger group of older adults (≥ 70 years) including data on cognitive function. We found that older Norwegian adults reported their number of teeth reasonably accurately compared to tooth counts from clinical examinations. The mean difference between self-reported and clinical number of teeth was low (− 0.22 teeth), and more than 70% of the participants reported their number of teeth within an error of two teeth. Correlation between self-reports and clinical examinations was high for the total sample. However, a lower correlation was found among participants with dementia, participants having ≥ 20 teeth, and participants with teeth restored or replaced by fixed prosthodontics. Moreover, almost all the older edentulous adults correctly reported having no teeth.

The reasonably good agreement between self-reported and clinical number of teeth found in study sample 1 is in line with recent studies on the validity of self-reported number of teeth [13, 14, 16, 23]. However, some limitations of the previous studies included that they had a smaller number of older adults than our study [13, 14, 23], were drawn from selected samples [14, 16, 23], or did not explore participants’ characteristics that could affect the accuracy of self-reported number of teeth [13, 16].

Consistently with previous studies [21, 23, 31], we found a tendency toward self-reported number of teeth being underreported compared to tooth counts from clinical examinations. In our study, the mean difference between the two measures was − 0.22 teeth. This low deviation between the two tooth count measures indicates that self-reported number of teeth can be a valid measure when used at a group level in epidemiological studies. This was further supported by our finding that more than 70% of the participants was within a self-report error of two teeth. Nonetheless, the 95% upper and lower limits of agreement in our study were wide and ranged from − 6.66 to 6.22 teeth, a finding that corresponds with Ueno’s study from 2018 [16]. The wide range in the upper and lower limits of agreement implies that caution should be taken when using self-reported number of teeth at an individual level.

In the present study, we also explored factors that might affect the validity of self-reported number of teeth among older people. In addition to sociodemographic factors, we addressed self-perceived general and oral health, cognitive function, number of teeth, and the complexity of the dental status assessed by the number of teeth restored or replaced by fixed prosthodontics. We found that impaired cognitive function, having ≥ 20 teeth, and a more complex dental status markedly decreased the accuracy of self-reported number of teeth.

Decline in the general counting ability is a known cognitive hallmark of Alzheimer’s disease [25]. However, to our knowledge, this is the first study to explore the impact of cognitive function on self-reported tooth count. In study sample 1, the correlation gradually decreased with impaired cognitive function with the lowest correlation in the group with dementia. These results emphasize that self-reported number of teeth in populations where dementia is prevalent should be interpreted with caution.

In study sample 2, about 80% of the participants had mild cognitive impairment or dementia. Despite the high prevalence of cognitive impairment, almost all participants correctly reported being dentulous or edentulous. This finding indicates that self-reports of having teeth or not is less influenced by cognitive impairment than self-reports of the exact number of teeth. Based on this we argue that self-reported edentulousness can be used as a valid oral health parameter even in older populations with a high prevalence of dementia.

Like previous studies [21, 23], we found that the accuracy of self-reported number of teeth was affected by increasing number of teeth recorded from the clinical examinations. Consistently with both Ueno et al. [21] and Matsui et al. [23], we found the self-reported number of teeth to be less accurate among those with ≥ 20 teeth compared to those with ≤ 19 teeth. We believe that the explanation for this is straightforward: a smaller number of teeth is easier to count and hence to self-report correctly.

In addition, we found that the self-report error increased with an increasing number of teeth restored or replaced by fixed prosthodontics (i.e., crowns, implants, bridge abutments and pontics). This is in line with previous studies, which have shown that a higher number of dental restorations and replacements reduces the accuracy of self-reported tooth counts [21–24]. Buhlin et al. observed that the difference between self-reported and clinical number of teeth was reduced when pontics were counted as natural teeth [24]. Similarly, Ueno et al. 2010 found that participants with fixed prosthetic pontics were more likely to incorrectly report their number of teeth than those with no pontics [21]. Furthermore, Matsui et al. reported the greatest discrepancy between self-reported and clinical tooth counts among those with many prosthetic teeth [23].

Root remnants is another factor that may affect the accuracy of self-reported tooth counts. Participants may not be aware of their root remnants or whether to include them in their self-reported number of teeth. We found that the underestimation of self-reported number of teeth increased when root remnants were included in the clinical number of teeth. This indicates that many root remnants were not counted in the self-reports. In a study by Axelsson, this was addressed by emphasizing not to count residual roots and fractured teeth in the questionnaire [32]. The findings of increased inaccuracy in self-reported number of teeth related to fixed prosthodontics and residual roots implies that future studies should include guidance on how to count present natural teeth.

Some limitations should be noted. Previous findings from the HUNT Study have shown that non-participants had somewhat lower socioeconomic status and slightly higher prevalence of chronic diseases and higher mortality than those who participated [33]. This may also apply to our study. Furthermore, in study sample 1, the oral health examinations were performed at field stations, which may have limited the participation of frail older adults. Lastly, as the HUNT Study was conducted in Norway, the findings may not be generalizable to populations with a different demographic and socioeconomic distribution.

The major strengths of our study were the large sample size of older adults and the population-based design. The sample was drawn from the general population in a Norwegian county with both small towns and rural areas. Additionally, the inclusion of a wide range of covariates, including cognitive function, enabled us to explore factors influencing the accuracy of self-reported number of teeth among older adults.

## Conclusions

We found an overall good agreement between self-reported and clinical number of teeth among older Norwegian adults (≥ 70 years). However, dementia, having ≥ 20 teeth, or fixed prosthetic restorations decreased the accuracy of self-reported number of teeth. Acknowledging these limitations, we consider that self-reported number of teeth is a valid measure at a group level among older adults in epidemiological studies when clinical examinations are not feasible. Furthermore, older adults accurately reported having no teeth, suggesting that edentulousness can be considered a valid self-report variable.

## Data Availability

The Trøndelag Health Study (HUNT) has invited persons aged 13–100 years to four surveys between 1984 and 2019. Comprehensive data from more than 140,000 persons having participated at least once and biological material from 78,000 persons are collected. The data are stored in HUNT databank and biological material in HUNT biobank. HUNT Research Centre has permission from the Norwegian Data Inspectorate to store and handle these data. The key identification in the data base is the personal identification number given to all Norwegians at birth or immigration, whilst de-identified data are sent to researchers upon approval of a research protocol by the Regional Ethical Committee and HUNT Research Centre. To protect participants’ privacy, HUNT Research Centre aims to limit storage of data outside HUNT databank and cannot deposit data in open repositories. HUNT databank holds precise information on all data exported to different projects and can reproduce these on request. There are no restrictions regarding data export given approval of applications to HUNT Research Centre. For more information see: www.ntnu.edu/hunt/data.

## Abbreviations

HUNT: The Trøndelag Health Study
ROAG-J: Revised Oral Assessment Guide-Jönköping
SD: Standard deviation
CI: Confidence interval
ULOA: Upper limit of agreement
LLOA: Lower limit of agreement
